# Beta-(1,3/1,6)-D-glucan from *Pleurotus ostreatus* in the prevention of recurrent respiratory tract infections: An international, multicentre, open-label, prospective study

**DOI:** 10.3389/fped.2022.999701

**Published:** 2022-10-14

**Authors:** Zuzana Rennerova, Leandro Picó Sirvent, Eva Carvajal Roca, Jarosław Paśnik, Mateja Logar, Katarina Milošević, Juraj Majtan, Milos Jesenak

**Affiliations:** ^1^ Department of Paediatric Pulmonology and Phthisiology, Faculty of Medicine, Slovak Medical University, National Children Institute of Health, Bratislava, Slovakia; ^2^ Paediatrics Department, Hospital de la Salud, Valencia, Spain; ^3^ Faculty of Medicine and Health Sciences, Valencia Catholic University Saint Vincent Martyr, Valencia, Spain; ^4^ Salve Medica Warszawa, Warsaw, Poland; ^5^ Department of Infectious Diseases, University Medical Center Ljubljana, Ljubljana, Slovenia; ^6^ Department of Infectious Diseases and Epidemiology, Faculty of Medicine, University of Ljubljana, Ljubljana, Slovenia; ^7^ Department of Pulmonology and Allergology, University Children’s Hospital, Belgrade, Serbia; ^8^ School of Medicine, University of Belgrade, Belgrade, Serbia; ^9^ Institute of Molecular Biology, Slovak Academy of Sciences, Bratislava, Slovakia; ^10^ Department of Microbiology, Faculty of Medicine, Slovak Medical University, Bratislava, Slovakia; ^11^ Department of Paediatrics, Jessenius Faculty of Medicine, University Teaching Hospital in Martin, Comenius University in Bratislava, Martin, Slovakia; ^12^ Department of Clinical Immunology and Allergology, University Teaching Hospital in Martin, Martin, Slovakia

**Keywords:** pleuran, respiratory tract infections, beta-glucan, children, tolerability

## Abstract

Preschool children are particularly susceptible to recurrent upper and lower respiratory tract infections due to their immune immaturity and other contributing factors. Preventing and/or treating children suffering from recurrent respiratory tract infections (RRTIs) is challenging, and it is important to provide more clinical evidence about the safety and efficacy of natural immunomodulating preparations, including β-glucans. The aim of the present study was to assess the incidence of respiratory tract infections (RTIs) in children with a history of RRTIs for a period of 6 months (3 months of pleuran supplementation and 3 months of follow-up) compared with the same period from October to March of the previous year prior to enrolment in the study. A total of 1,030 children with a mean age of 3.49 ± 1.91 years from seven countries were included in this study. The total number of RTIs observed during the study period was significantly lower compared to the same period of the previous year (7.07 ± 2.89 vs. 3.87 ± 3.19; *p* < 0.001). Analysis of each type of RTI revealed significant reductions in the mean number and duration of infections for all RTI subtypes compared to the previous year. This study also confirmed the beneficial safety profile of pleuran supplementation. In conclusion, pleuran supplementation represents an interesting and prospective supplement in preventing respiratory infections and reveals new strategies for supporting immune functions in the paediatric population.

## Introduction

Recurrent respiratory tract infections (RRTIs) are very common in infants and children and are characterised by recurring upper and lower respiratory tract infections, mainly in school-age and preschool children (1). RRTIs are defined as a minimum of six to eight episodes per year, affecting 15%–20% of children under 5 years of age (2). The incidence of RRTIs has been increasing in recent years due to deterioration caused by environmental pollution and changes in lifestyle (e.g., early socialisation of children) (3).

A recent population study attempted to characterise the major potential risk factors of RRTIs in preschool children (4). In addition to the traditional risk factors of RRTIs (e.g., older siblings, bigger family size, day-care attendance centres), asthma has been identified as a leading risk factor, followed by allergy/atopy, non-indicated use of antibiotics and shortage of breastfeeding. All characterised risk factors might have a dose-dependent effect on RRTI susceptibility in preschool children. The pathogenesis of RRTIs is proposed to be multifactorial and complex due to the variability of microbial causes, immune immaturity and respiratory diseases (5).

There are several approaches to prevent and/or treat children suffering from RRTIs using immunomodulating preparations, biologically active polysaccharides, probiotics and vitamins, as well as complementary and alternative medical products, such as herbal and bee products (5). Immunostimulants, including pidotimod and bacterial lysates, attract much attention and have become an object of several clinical studies and meta-analyses. Indeed, a very recent meta-analysis, which included 14 articles with 2,400 paediatric subjects, showed that pidotimod and OM-85 BV (bacterial lysate) effectively reduced the incidence of RRTIs in children (6). However, the authors of the meta-analysis highlighted some limitations of the analysed clinical studies; therefore, the conclusion of the meta-analysis needs to be interpreted with caution.

A recently presented international consensus on products used for the prevention of respiratory tract infections (RTIs) in susceptible children, including those that are atopic/allergic or asthmatic, came to the conclusion that, among the analysed approaches/products, only OM-85 has a sufficient number of well-conducted clinical trials with an adequate safety profile (5). According to experts' statements from the World Association of Infectious Diseases and Immunological Disorders, probiotics, β-glucans, vitamins, echinacea and several other bacterial lysates are not recommended for the prevention of recurrences in RTI-susceptible children. The major limitation of non-recommended approaches is the absence of robust clinical studies with high methodological quality. However, the authors stated that several clinical trials indicated a potential of, e.g., β-glucans in the prevention of RRTIs and new trials and studies are highly needed.

Despite the fact that most of these approaches to the prevention of RRTIs in children are not yet recommended, they could play a beneficial role in children with RRTIs. One of the promising candidates in preventing RRTIs with a good safety and tolerability profile are β-glucans (biologically active polysaccharides). Various β-glucans of different origins have been shown to possess interesting biological activities, including anti-inflammatory and immunomodulatory activities. Although the basic structures of β-glucans from different sources are almost identical, they often show differences in bioactivity (7).

Regarding RRTIs in the paediatric population, the most clinically studied β-glucan is insoluble beta-(1,3/1,6)-D-glucan from *Pleurotus ostreatus* (pleuran), which has been administered especially in syrup form (8–11). One of the major weaknesses of clinical studies performed with pleuran is the small number of enrolled paediatric subjects and the fact that there was a different duration period of pleuran administration.

Therefore, in this international, multicentre, prospective, open-label and follow-up study, conducted in 89 paediatric departments and practices in seven European countries (Spain, Poland, Slovenia, Croatia, Bosnia and Herzegovina, Serbia, and Turkey) between 2011 and 2018, we aimed to assess the incidence of respiratory infections in children with a history of RRTIs for a period of 6 months (from October to March), after 3 months of supplementation with a food supplement based on pleuran and 3 months of follow-up, compared with the same period of the previous year prior to enrolment in the study. Furthermore, the tolerability and safety of β-glucan supplementation were evaluated.

## Methods

### Patients

This study included children between the ages of 1 and 12 years who visited their paediatrician during the months from October to November and had a documented history of RRTIs during the period between October and March of the previous year. The enrolment of the children was based on the adapted definition of RRTIs by De Martino et al. (12): for younger children (<4 years) more than 6 upper or lower respiratory tract infections and for older children (≥4 years) more than 4 respiratory tract infections of the upper or lower respiratory tract during the period from October to March in the previous year.

The following exclusion criteria were used: birth before week 34, mechanical ventilation in the neonatal period, bronchopulmonary dysplasia, primary immunodeficiency syndromes, cystic fibrosis, chronic diarrhoea and intolerance to any of the ingredients of the product. Children who were treated with other immunomodulators or some medications for the prevention or treatment of RTI symptoms in the 15 days prior to enrolment (corticosteroids, montelukast, antibiotics, homoeopathy, inosine pranobex, bacterial lysates, etc.) were excluded. Children whose parents/tutors disagreed with participation were also excluded.

### Study design

This open-label study consisted of a 3-month period of supplementation of Imunoglukan P4H® liquid (10 mg of pleuran and 10 mg of vitamin C in 1 ml of liquid) and a 3-month follow-up period between October and March with three clinical visits (V1 at the beginning, V2 after 3 months of supplementation and V3 after 3 months of follow-up). The children that met the inclusion criteria were required to take Imunoglukan P4H® liquid at a dose of 1 ml per 5 kg of body weight every morning on an empty stomach during a period of three months. The active substance of the administered natural product is a complex of biologically active polysaccharides that consists in beta-1,3/1,6-D-glucan pleuran isolated from edible mushroom *Pleurotus ostreatus* by patented technology. It was previously identified and chemically characterised by Karacsonyi and Kuniak (13).

At the time of inclusion in the study, each participating paediatrician completed the questionnaire and collected data on relevant family and perinatal history, anthropometric data and data on breastfeeding, tobacco smoke exposure, residence in urban areas and nursery or school attendance. During 6 months of treatment and follow-up period, the number and subtype of RTIs (common cold, otitis media, pharyngotonsillitis, laryngitis, bronchitis, pneumonia) were recorded and compared with the incidence and the number of RTI episodes reported during the same period between October and March of the previous year. In Slovenia, Croatia, Bosnia and Herzegovina, and Serbia, they also registered and compared the duration of RTIs, both the total duration of infections and the duration of each subtype of infection. Secondary endpoints that were monitored throughout the study period included product tolerability and occurrence of adverse events. At the end of the study, the opinions of parents/tutors and paediatricians about product acceptance (3-very good, 2-good, 1-regular, 0-poor) and the perception of clinical improvement (as being “better,” “the same,” or “worse”) were recorded. The study excluded children who took the study product irregularly (less than 75% of the prescribed doses).

### Statistical analyses

Discrete data are presented using frequency tables *n*/*N* (%). Continuous data are presented as n, mean, SD, minimum, maximum and median. The differences between groups of patients are tested by chi-squared tests in the case of discrete data and *t*-tests and ANOVA in the case of continuous data. The tests are two-sided with significance considered at *p* < 0.05. There was no correction due to multiple testing, and the *p*-values were presented in the exploratory fashion. Statistical analyses were performed using SPSS 19.0 software for Windows (SPPS Inc., Chicago, IL, USA).

## Results

A total of 1,030 children from seven countries were included in this study, of which 994 children (96.5%) met all the requirements of the study protocol and were considered valid for the per protocol analysis. The mean age of children who completed the protocol was 3.49 ± 1.91 years, and 61.9% were younger than 4 years. Most of the subjects (57.3%) were male. The demographic characteristics of the analysed subjects are presented in Table 1.

**Table 1:** General characteristics of the study population according to country.

Country	Patients enrolled (*n*)	Patients analysed (*n*)	Male [*n*, (%)]	Mean age (year)	Patients proportion in study (%)
Serbia (SRB)	311	302	176 (58.3)	3.34	30.38
Poland (PL)^a^	194	191	120 (62.8)	3.70	19.22
Spain (ESP)^a^	166	151	85 (56.3)	3.01	15.19
Bosnia and Herzegovina (BIH)	120	117	59 (50.4)	3.85	11.77
Slovenia (SLO)	109	107	58 (54.2)	3.34	10.76
Croatia (CRO)	100	98	58 (59.2)	3.37	9.86
Turkey (TR)	30	28	14 (50.0)	7.14	2.82

^a^ Data of these cohorts were published separately (10, 11).

**Figure 1:**
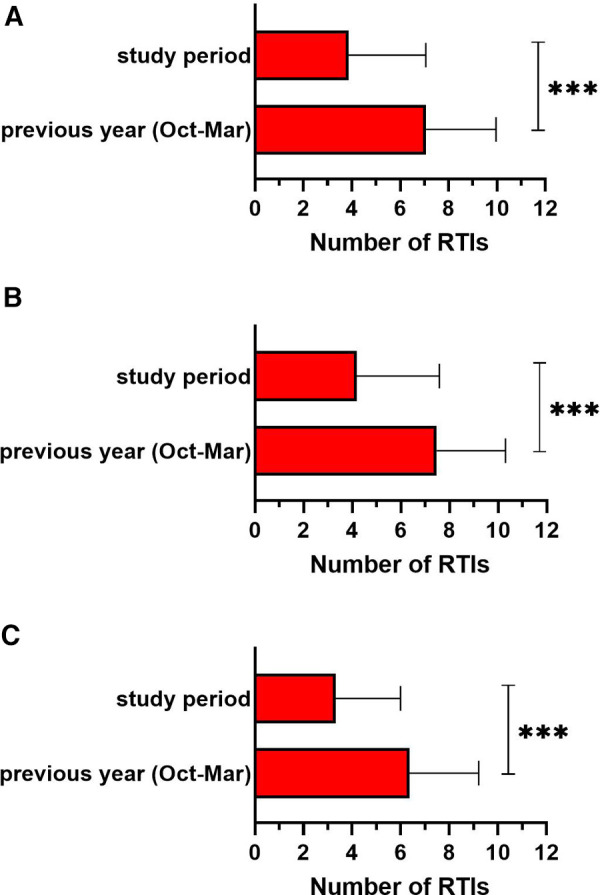
The total number of respiratory tract infections (RTIs) in the study period compared to the same period of the previous year (october–march) in (**A**) all children (*n* = 994), (**B**) in children under 4 years old (*n* = 609) and (**C**) in children over 4 years old (*n* = 385). ***p < 0.001.

The total number of RTIs observed during the study period was significantly lower compared to the same period of the previous year (7.07 ± 2.89 vs. 3.87 ± 3.19; *p* < 0.001). We observed a 45.3% decrease in total infections. A comparable statistically significant decline was observed in both age groups: in children under 4 years of age (7.47 ± 2.84 vs. 4.18 ± 3.41; *p* < 0.001) and in older children over 4 years of age (6.36 ± 2.85 vs. 3.32 ± 2.67; *p* < 0.001) (Figure 1).

### Subtypes of respiratory tract infections

Analysis of each type of RTI revealed significant reductions in the mean number of infections for all RTI subtypes compared to the previous year. The mean number of infections was reduced by 25.1 to 61.0%, depending on their subtype (Table 2). Comparable statistically significant declines were found in both age groups (<4 years and ≥4 years).

**Table 2:** Incidence of RTI subtypes in all subjects (*n* = 994) in the study.

RTI subtype	Number of RTIs			
Previous year (oct–march)	Study period (Oct–March)	*p*-value	Reduction of RTIs^a^ (%)
Otitis	1.26 ± 1.28	0.53 ± 0.96	<0.001	57.8
Common cold	2.38 ± 1.63	1.79 ± 1.80	<0.001	25.1
Tonsillopharyngitis	1.56 ± 1.47	0.76 ± 1.10	<0.001	51.6
Laryngitis	0.53 ± 0.92	0.21 ± 0.65	<0.001	61.0
Bronchitis	1.15 ± 1.44	0.49 ± 0.99	<0.001	57.2
Pneumonias	0.18 ± 0.57	0.09 ± 0.44	<0.001	49.1

^a^ Reduction in the number of RTI subtypes during the study period in comparison with the same period of the previous year (October–March).

### Duration of respiratory tract infections

**Figure 2:**
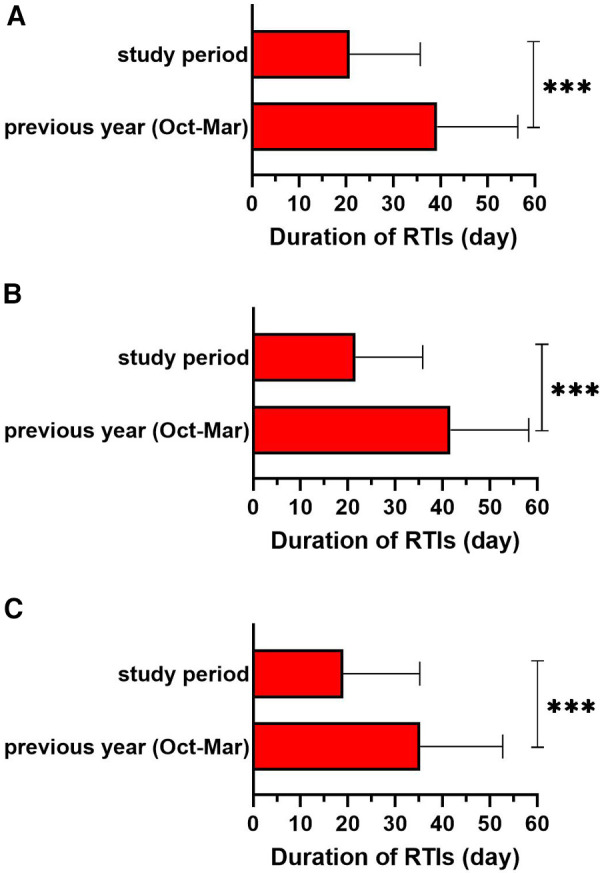
Sub-analysis of duration of respiratory tract infections (RTIs) in the study period compared to the same period of the previous year (october–march) in (**A**) whole subgroup of children (*n* = 624), (**B**) in children under 4 years old (*n* = 390) and (**C**) in children over 4 years old (*n* = 234). Data from Slovenia, Croatia, Bosnia and Herzegovina, and Serbia were analysed. ***p < 0.001.

In Slovenia, Croatia, Bosnia and Herzegovina, and Serbia, an additional analysis of the total duration of RTIs was carried out. The sub-analysis showed that the mean duration of RTIs was significantly shorter during the study period compared to the same period of the previous year (39.3 ± 17.1 vs. 20.7 ± 15.0; *p* < 0.001) (Figure 2). The duration of RTIs was reduced by 25.0 to 66.1%, depending on their subtype, and 47.4% for total infections (Supplementary Table S1).

### Safety and tolerability

In the safety analysis, nine non-serious adverse events were recorded, of which five cases had a possible link to the study product and three of them led to early discontinuation upon parent's discretion. Of parents/tutors, 97.3% reported good or very good acceptance of the product (except four children who were excluded from the study due to product taste intolerance and its refusal), while 93.0% of them considered that the health status of their children improved during the study period compared to the previous year.

## Discussion

RTIs represent the most common form of infection in all age categories. RRTIs are a special form of airway infection typically presented in early life and are associated with a significant impact on the quality of life of affected subjects and their families. Therefore, all proven tools for RRTI prevention are highly appreciated and needed. Entering the complex process of immune system development by external stimuli (natural or synthetic) should be personalised and very careful. Natural immunomodulators are used as the first line of immune support, especially in the context of their safety. β-glucans are one of the most studied and used natural substances with many beneficial biological activities that are also useful for immune support and RRTI prevention.

Based on the previous results from smaller groups of children in Spain (10) and Poland (11), we analysed the safety and efficacy of pleuran in more countries in which we performed a study of the same design. In a cohort of 1,030 children from various European countries with different geographical settings and other specific characteristics, we analysed the clinical efficacy and tolerability of pleuran on the incidence and duration of various forms of upper and lower airway infections. In general, we confirmed the significant efficacy on the prevention of various forms of RTIs expressed by the decline in the total number of infections, their particular type and their duration in comparison with the data from the same period of the previous year. Sub-analysis of additional data obtained from Spain (10) also revealed significant reductions in other variables (e.g., absence from nursery/school, emergency service visits, necessity for pharmacological treatment). In Poland, reduction of the number of missed school days compared to the same period of the previous year was evaluated with statistical significance (11). The studied natural compound was safe, and no serious adverse events were recorded. Most of the parents reported good or very good acceptance of the product and improvements in the health status of their children.

RRTIs in children are associated with various direct and indirect impacts on the quality of life of the affected children as well as of their parents. β-glucans represent a prospective and safe tool for preventing the occurrence of RTIs and decreasing their severity and subsequent complications. The therapeutic strategy should focus on the use of effective preventive substances with confirmed efficacy and safety from clinical trials. Moreover, the use of effective preventive measures could substantially decrease the overuse of antibiotics. The clinical efficacy and preventive capacity of β-glucans in respiratory tract infections was studied in various age groups and subjects (e.g., children, athletes, healthy adults, chronically ill patients). Systematic reviews and meta-analyses have confirmed the potential role of β-glucans in human respiratory tract infections; however, the analysed studies showed high heterogeneity (5, 14, 15).

In children, the application of pleuran from oyster mushrooms significantly reduced the number of RTIs in most children, both in open-label trials (8, 10, 11) and a double-blind placebo-controlled trial (9). Moreover, treatment with pleuran induced favourable immune changes and was well tolerated (9). In the present study, the same active compound decreased both the frequency and duration of RTIs. A significant impact was observed on both upper and lower airway infections. A similar effect on respiratory health and laboratory parameters was also observed in several trials with yeast-derived β-glucans (16, 17). Similarly, in a recent clinical trial with the combined immunomodulating preparation (containing yeast-derived β-glucan, zinc, vitamin D3 and extract from *Sambucus nigra*) decreased the number of upper and lower respiratory infections and their duration (18).

Several studies have aimed to verify the possible effect of β-glucans on the RTIs in adult patients, who usually do not suffer from such a high frequency of infections compared to children. However, in risky situations (immune suppression, chronic diseases, elite athletes, periods with increased psychic stress, etc.), the risk of infection could be significantly increased. β-glucans from *Euglena gracilis* decreased the number of sick days, upper RTI symptoms and lower global severity in healthy active adults (19). Similar results with yeast β-glucans were recently confirmed in healthy adults. Furthermore, the clinical effect was accompanied by a positive effect on blood pressure and improvement of mood (20). Another interesting area of clinical application of β-glucans is immune protection against the immunosuppression associated with intensive sport efforts. Pleuran decreases the incidence of upper respiratory tract infection (URTI) symptoms and prevents the decline of immune function in elite athletes (21, 22). Dispersible yeast-derived β-glucan contained in beverages decreased the frequency and severity of URTIs and significantly reduced the average missed post-marathon workout days in marathon runners (23). Interestingly, the solubility of the applied β-glucans can yield various effects on respiratory infections. Another study with marathon runners, the insoluble yeast β-glucan group but not the soluble yeast β-glucan group reported fewer URTI symptomatic days and lower severity of URTI than the placebo group (24).

The mode of action of β-glucans was studied and revealed by many laboratory experiments, animal models and clinical studies. The most important receptor mediating the biological effects of β-glucans is dectin-1, which is expressed on many immune and non-immune cells (25). These natural immunomodulators showed the capacity to modulate both innate and specific immunity with direct and indirect impacts on cell phenotypes, activity and cytokine microenvironment composition (26). A recent animal experiment with β-glucan extract from *Lentinus edodes* (Lentinan) confirmed the cytoprotective effect on pulmonary cells and anti-inflammatory activity expressed by the changes in the cytokine spectrum. Furthermore, lentinan showed a high degree of protection against infection with the influenza A virus in mice (27). It alleviated the pathological changes of infected mice and inhibited inflammatory cytokine levels in the serum and lungs *via* regulation of the TLR4/MyD88 signalling pathway. These results could support the use of β-glucans in the management of COVID-19 (28). Another important and useful mode of action could be the induction of trained immunity (29). The potency of immunomodulatory effects differs among the various sources of β-glucans and the branching of their molecule. A (1,6)-beta-linked side chains in the molecule of mushroom- and yeast-derived β-glucans were shown to be essential for their immunomodulating effects (30). Most of the studies with positive preventive effects on respiratory morbidity were performed either with β-glucans from mushroom (especially pleuran from *Pleurotus ostreatus*) or yeasts. The results with oat-derived β-glucans were inconclusive and non-significant (31, 32).

The presented study has several strengths but also a few limitations. First, the open label design has well-known problems and biases (especially the absence of a placebo-treated group); however, the high number of enrolled children (from the age of 1 year) and the small number of early terminations support the positive clinical observations. Second, pleuran was not used by itself but was used in combination with vitamin C. The interaction between different β-glucans and vitamin C has already been characterised in animal models and the combination results in augmentation of innate immune responses in fish (33, 34). This combination may therefore provide the beneficial synergic and complementary immunomodulatory activity. However, the knowledge about the beneficial synergic effect of this combination in humans is limited (9) and warrants further research.

On the other hand, the homogeneity of the achieved results across the various countries with different climates and geographical settings represents another strength of the study. Significant agreement on the positive evaluation by parents and paediatricians is also of special importance. This study also confirmed the beneficial safety profile without new warning signs for the clinical use of this natural compound.

In conclusion, RRTIs represent a substantial and specific clinical issue in daily paediatric praxis. Their impact on the health status and quality of life of affected children and their families is challenging. β-glucans represent an interesting and prospective way to prevent new episodes of respiratory infections and support immune functions. This study confirmed the significant efficacy of pleuran from oyster mushrooms on the prevention of various forms of RTIs in both age groups of children (under and above 4 years of age) in comparison with the data from the same period (from October to March) of the previous year. Pleuran has been shown to be well tolerated and has a favourable safety profile at the dose level used in this study (10 mg of pleuran and 10 mg of vitamin C per 5 kg of body weight).

## Data Availability

The raw data supporting the conclusions of this article will be made available by the authors, without undue reservation.
