# Supplementary material for: Beta-(1,3/1,6)-D-glucan from Pleurotus ostreatus in the prevention of recurrent respiratory tract infections: An international, multicentre, open-label, prospective study
Source: Front Pediatr. 2022 Oct 14;10:999701. doi: 10.3389/fped.2022.999701 (PMC9619242; doi:10.3389/fped.2022.999701)
Supplement: Supplementary file 1 [file Table1.docx]

Supplementary Material

**Table S1.** Duration of RTI subtypes in subgroup of subjects (n = 624) in the study.

| **RTI subtype** | **Duration of RTIs**** | | |  | |
| --- | --- | --- | --- | --- | --- |
|  | **Previous year**  **(Oct–March)** | **Study period**  **(Oct–March)** | ***P*-value** | **Reduction of RTIs***  **(%)** | |
| otitis | 8.8 ± 9.7 | 3.8 ± 6.5 | < 0.001 | | 57.2 |
| common cold | 10.6 ± 9.0 | 8.0 ± 8.6 | < 0.001 | | 25.0 |
| tonsillopharyngitis | 9.3 ± 10.5 | 4.8 ± 6.6 | < 0.001 | | 49.1 |
| laryngitis | 2.4 ± 4.8 | 1.0 ± 3.7 | < 0.001 | | 58.1 |
| bronchitis | 7.1 ± 10.5 | 2.8 ± 5.9 | < 0.001 | | 59.9 |
| pneumonia | 1.1 ± 3.5 | 0.4 ± 2.1 | < 0.001 | | 66.1 |

* Reduction in the duration of RTI subtypes during the study period in comparison with the same period of the previous year (October-March).

** Data from Slovenia, Croatia, Bosnia and Herzegovina, and Serbia were analysed.
